# Mr. Cornish's Case of Croup

**Published:** 1813-04

**Authors:** James Cornish


*To the Editors of the Medical and Physical Journal.*

GENTLEMEN,

IF you think the following case of Croup worthy a place in your valuable Journal, it is very much at your service.

Your humble Servant,

JAMES CORNISH.

James Earle, ætat. two years, of a robust constitution, was, on the 21st of January last, attacked with croup. The difficulty of breathing was very great, particularly of inspiration, which was accompanied with that peculiar stridulous sound for which this disease is remarkable. He had a troublesome dry cough, and a considerable degree of fever. The pulse was so quick that it could not be counted. The child had been unwell for two days before this, and had some little hoarseness; but, as his appetite was not at all impaired, and he took his rest well, the parents thought but little of it. The symptoms had now alarmingly and suddenly increased. Nine leeches were applied over the lower part of the trachea, and he took an emetic of two grains of tartrate of antimony. As this operated but slightly, half a grain more was given every half hour for three successive hours, and this vomited and purged him freely. The difficulty of respiration, after the operation of vomiting, was lessened considerably, and the ringing sound on inspiration almost lost; the bleeding of the leeches was encouraged for several hours, and his feet immersed in hot water.

On

On the following day (22d) the difficulty of breathing had increased, he had passed a sleepless night, had great thirst, and the violence of the febrile symptoms was not at all abated. One grain of antimonial powder, with an equal quantity of submuriate of mercury, was given him every four hours; and eight leeches more were applied over the upper part of the chest. In the evening the dose of antimony and mercury was doubled, and given at the interval of two hours; a large blister was also laid on.

23d.—The lymph which had exuded from the trachea and larynx, became so much thickened, that the efforts to breathe were very distressing: to relieve this, the steam of hot water was inhaled for an hour or more. The restlessness was extreme, and the skin dry and hot. The dose of the medicine was increased to three grains of each every two hours. This did not make the child sick throughout the day, and the bowels were opened but once; the fœces were very hard and black. In the evening the dose of antimonial powder was increased to four grains, and the calomel reduced to two, still given at the same interval of two hours.

24th.—The breathing was not at all relieved, and he had been very restless all night. He had one stool, which was very green; and nauseated a little, but brought nothing off. The pulse was quick, as at first; the thirst was very great, with considerable febrile heat, and dry skin. Two grains of calomel, one of powdered digitalis, and half a grain of tartrate of antimony, were given every six hours. This vomited him in the course of the day, and occasioned the separation of a quantity of filmy or ropy mucus. He had one stool of a natural color, and by the evening the difficulty of breathing was much lessened.

25th.—By this time the breathing was greatly relieved, and he had slept some hours; the nausea was continued during the night, and more of the stringy deposit had been thrown off from the trachea. The cough was much looser, the pulse nearly natural, and an equal moisture was diffused over the skin. He had now taken four grains of the digitalis. From the exhibition of the first emetic, the stridulous sound was nearly altogether lost. Half a grain of tartrate of antimony was now given in almond emulsion every three hours; the first dose made him sick; he coughed up a considerable quantity of the membranous film, which he swallowed, and this was brought off by stool nearly in the same state.

26th.—The nausea had been urgent at times, the bowels had been moved, and he slept well. His respiration was comparatively free, and his pulse rather quicker than yesterday.

terday. The medicine was continued, and by the evening the oppression in breathing was hardly to be noticed. He slept well that night, and has had no return of any unpleasant symptom. He has since taken the almond emulsion, with one-eighth of a grain of emetic tartar, when the cough was troublesome.

I am not aware that any writers have particularly noticed the torpid state of the stomach and bowels in croup. That such state existed to a great degree in the preceding case, no one will doubt, when he considers the comparatively slight effect produced by the large doses of calomel and antimony which were given. In the space of about forty-eight hours, forty-nine grains of antimonial powder were taken; and, in less than three days, forty-seven grains of calomel: yet the bowels were never opened more than twice any day during the disease; the salivary glands were not at all affected; nor was the breath in the least tainted. It has been said, "to excite salivation is to effectually cure the disease;" but, where there is such insensibility to the operation of such powerful medicines, salivation will be difficultly excited. That this state of torpor universally exists in croup, I am inclined to believe, from the assurances of a gentleman* of great respectability, who has practised in this town with considerable success upwards of twenty years; and who, during that time, has seen more than thirty cases of the disease, in every one of which he has not failed to remark it. Contrary to the opinion that this disease is peculiar to children, he has also informed me that he has seen two decided cases of adult croup. He is inclined to believe it contagious, but in no state spasmodic; what has been averred to be spasm, being the exacerbation of remitting fever. From the frequency of the disease in this town, which is nearly surrounded by the sea, it would appear to be most prevalent near the sea-coast.

I beg leave to notice the powerful effect of a timely emetic, in some cases instantly removing the complaint. Three children, who had been exposed uncovered to damp evening air, were suddenly seized with the disease. They had been put to bed to all appearance in perfect health; but, some time after, the parents were alarmed at the noise they made in breathing. The sonorous inspiration immediately evinced the nature of the affection. A strong emetic of tartrate of antimony was given to each of them, which completely succeeded in overpowering it.

*Falmouth*, *Feb.* 3, 1813.

---

* Mr. Trevosso.

